# In silico analysis of CSF2RB from cancer genomic databases reveals a heterogeneous role in different breast cancer subtypes

**DOI:** 10.3389/fbinf.2025.1606828

**Published:** 2025-08-05

**Authors:** Raghad Alshelaiel, Abdulmohsen Alkushi, Lolwah Abdullah Alriyees, Abir Abdullah Alamro, Humidah Alanazi, Areej Alhareeri, Bader AlMuzzaini, Mamoon Rashid

**Affiliations:** ^1^ Department of Biochemistry, College of Science, King Saud University, Riyadh, Saudi Arabia; ^2^ College of Science and Health Professions, King Saud bin Abdulaziz University for Health Sciences, Riyadh, Saudi Arabia; ^3^ King Abdullah International Medical Research Center, Riyadh, Saudi Arabia; ^4^ Department of Surgery, King Abdulaziz Medical City, MNGHA, Riyadh, Saudi Arabia; ^5^ Clinical Laboratory Sciences Department, College of Applied Medical Sciences, King Saud Bin Abdulaziz University for Health Sciences, Riyadh, Saudi Arabia; ^6^ Department of Medical Genomics, King Abdullah International Medical Research Center, Riyadh, Saudi Arabia; ^7^ King Saud bin Abdulaziz University for Health Sciences, Riyadh, Saudi Arabia; ^8^ Department of AI and Bioinformatics, King Abdullah International Medical Research Center, Riyadh, Saudi Arabia

**Keywords:** cancer, breast cancer, colony-stimulating factor 2 receptor subunit beta, prognosis, cancer genomics, bioinformatics

## Abstract

**Objective:**

*CSF2RB* is the common beta chain of the heterodimeric receptors for the cytokines, granulocyte–macrophage colony-stimulating factor (GM-CSF), interleukin 3 (IL-3), and interleukin 5 (IL-5). The activation of these cell surface receptors results in functional responses including cellular proliferation, differentiation, survival, and maturation via multiple signaling pathways such as JAK2/STAT5, MAPK, and PI3-kinase/AKT. Moreover, *CSF2RB* is abnormally expressed in a variety of tumors, especially in leukemia. The implications of *CSF2RB* in breast cancer remain unclear and have not been widely studied.

**Methods:**

We analyzed *CSF2RB* genetic changes, mRNA expression, DNA methylation, prognosis, and immune infiltration levels across different tumor types, with a focus on breast and hematological malignancies. The data used in this study were obtained from publicly available cancer genomics databases, such as TCGA, cBioPortal, TIMER2.0, GEPIA, and UALCAN.

**Results:**

Our *in silico* analyses showed overexpression of *CSF2RB* in acute myeloid leukemia (AML) and decreased expression in breast invasive carcinoma (BRCA) compared to matched normal samples. Promoter methylation of *CSF2RB* was elevated in BRCA samples compared to normal samples. Our analysis further demonstrates that the *CSF2RB* gene has a favorable prognostic effect in BRCA, although this was not statistically significant across all databases studied. We found that BRCA and its subtypes exhibit high CD8^+^ T-cell infiltration levels that are positively correlated with the *CSF2RB* gene expression level. Wild-type *CSF2RB* shows higher expression than the mutated *CSF2RB* in breast cancer. *CSF2RB* expression (and/or mutation) has no significant effect on the overall survival probability. *CSF2RB* expression is downregulated in luminal and HER2-positive samples but upregulated in triple-negative breast cancer (TNBC), compared to that in normal samples.

**Conclusion:**

The results suggest a diverse role for the *CSF2RB* gene across different subtypes of breast cancer. To attribute a clear role to *CSF2RB* in breast cancer, further functional studies focusing on differential gene expression, methylation, and their prognostic effect in each breast cancer subtype are required.

## Introduction

β common (βc) cytokines are produced by various cell types in the human body and can exert their effects either locally—in the vicinity of cells that produce them—or at distant sites in the body. The colony-stimulating factor 2 receptor subunit beta (*CSF2RB*) gene encodes CD131, the common beta chain of the heterodimeric receptors for β common cytokines: granulocyte–macrophage colony-stimulating factor (GM-CSF), interleukin 3 (IL-3), and interleukin 5 (IL-5). These receptors are heterodimers composed of a common beta chain and distinct alpha chains, with the alpha chains conferring cytokine specificity. The ligand-specific alpha chain (CSF2RA) forms a receptor complex with the common beta chain (*CSF2RB)* and its ligand (GM-CSF), creating a dodecameric complex. Within this structure, the close presence of *CSF2RB* subunits enables the associated JAK2 kinases to trans-phosphorylate. Ligand binding to the cell surface receptors for GM-CSF, IL-3, and IL-5 activates these receptors and triggers functional responses, including cellular proliferation, differentiation, survival, and maturation. Although the exact process of receptor activation remains unclear, ligand-induced dimerization of receptor subunits appears to be a crucial step in the signaling process. Pleiotropic cytokines such as IL-3, IL-5, and GM-CSF are known to promote hematopoiesis and regulate the growth and activity of immune cells (Freeburn et al., 1998; Hercus et al., 2018; Côrte-Real et al., 2022; Watanabe-Smith et al., 2016).

Multiple signaling pathways are initiated when the cytokine–receptor complex assembles in a specific stoichiometry, which is triggered by cytokine binding. These include interactions with various receptor systems and activation of the Janus kinase (JAK) family and STAT5 pathway, the mitogen-activated protein kinase (MAPK), and the phosphoinositide 3-kinase (PI3K) pathways. These signaling cascades lead to numerous biological responses, such as cell survival, proliferation, differentiation, migration, and the effector functions of mature leukocytes (Côrte-Real et al., 2022).

There are differences between GM-CSF, IL-3, and IL-5. GM-CSF is secreted by multiple cell types, including macrophages, fibroblasts, and epithelial cells, while IL-3 and IL-5 are secreted by T-cells. Cytokines play various roles in innate and adaptive immune responses and inflammatory diseases. It is becoming more widely acknowledged that the common beta chain *CSF2RB* cytokines are involved in the pathogenesis of various types of leukemia. GM-CSF functions as a growth and survival factor in human myeloid leukemia and improves the prognosis of chronic myeloid leukemia (CML) (Hercus et al., 2018). Acute myeloid leukemia (AML) patients’ blast cells exhibit increased GM-CSF receptor expression and proliferation. Additionally, IL-3 modulates megakaryocytes and hemopoietic stem cells, and it serves as a growth and survival factor for several lineages of both normal and malignant hemopoietic cells. Based on a recent article (Watanabe-Smith et al., 2016), a patient with T-cell acute lymphoblastic leukemia (T-ALL) presented a germline mutation in the *CSF2RB* gene, which can induce cell proliferation *in vitro*. Early evidence establishing the role of *CSF2RB* mutations in hematological cell lines and malignancies appeared at the end of the 20th century (D'Andrea et al., 1994; Jenkins et al., 1996; Jenkins et al., 1995).

Charlet et al. (2022) investigated the role of *CSF2RB* in FMS-like tyrosine kinase 3 receptor (FLT3)-internal tandem duplication (ITD)-positive AML through *in vitro* and *in vivo* studies and confirmed *CSF2RB*/FLT3-ITD interaction in human primary leukemic cells and cell lines with FLT3-ITD mutation. The ITD mutation found in one-third of AML cases is a mutation of FLT3 leading to receptor activation and downstream signal transduction. Binding of *CSF2RB* to FLT3-ITD contributes to FLT3-dependent STAT5 activation, promoting oncogenic signaling and the transformation of AML *in vitro* and *in vivo*. FLT3 forms a complex with *CSF2RB*, leading to its phosphorylation and activation. Activated *CSF2RB* leads to STAT5 phosphorylation, which then mediates the oncogenic transformation of AML. Charlet et al. (2022) knocked down *CSF2RB* in AML cell lines with FLT3-ITD mutation and also transfected *CSF2RB*-deficient mice with FLT3-ITD mutation. The result was decreased STAT5 phosphorylation, reduced cell proliferation, and increased FLT3 inhibition sensitivity. In addition, the onset of the disease was delayed in the transfected mice, and there was an increase in the survival rate. They concluded that *CSF2RB* can be used to treat AML with FLT3-ITD mutation by discovering therapeutic peptides to target and interfere with FLT3-ITD-dependent *CSF2RB* activation (Charlet et al., 2022).

Interest in the function of βc cytokines outside the hematopoietic lineages has grown in recent years. It has been found that the expression of GM-CSF is linked to some tumors, such as colorectal cancer (Li et al., 2016), neuroblastoma (Agarwal et al., 2015), and breast cancer (Park et al., 2011). Moreover, it has been shown to induce angiogenesis and local tumor invasion in head and neck cancer (Lopes-Santos et al., 2023). Numerous investigations have also revealed that myeloid-derived suppressor cells (MDSCs), which are activated by GM-CSF, play a role in the metastasis of glioma, pancreatic, and liver malignancies. In addition, IL-3 could potentially play a pathogenic role in some solid tumors. Endothelial cells, endothelial progenitor cells, and tumor-derived endothelial cells all express IL-3 receptors, which promote angiogenesis and tumor progression. A study (C Bonder and Lopez, unpublished) implied that IL-3 and its receptor may be targeted to inhibit the growth and activity of some breast cancer cells (Hercus et al., 2018).

Breast cancer accounts for one in eight cancer cases and is the leading cause of cancer-related deaths among women, with its incidence dramatically increasing in recent years. The World Health Organization (WHO) and the International Agency for Research on Cancer (IARC) reported 2.26 million new cases of breast cancer and 684,996 cases of breast cancer-related deaths in women worldwide in 2020. Furthermore, it is estimated that by 2040, there will be more than 3 million new cases diagnosed and 1 million deaths due to breast cancer (Sung et al., 2021; Arnold et al., 2022). Currently, radiographic scanning, clinical/pathological testing, physical examination, and histological data are used to classify and diagnose breast cancer. Molecular diagnostics are not frequently used in clinical settings, although they provide an accurate, equitable, and effective means of classifying breast cancer. The use of gene expression profiling to detect variations in gene activity offers a novel and accurate approach to diagnosing breast cancer (Mirza et al., 2023). Subsequently, an array of gene expression signatures has been documented for diverse cancers, serving to categorize tumors, tumor types, and stages and predict the prognosis of the cancer. Breast cancer is a very heterogeneous disease; it is still difficult to identify a single set of biomarkers or a gene signature that is common to all molecular subtypes (Mirza et al., 2023).

Rashid et al. (2021) identified a somatic, potentially transforming, and oncogenic *CSF2RB* mutation (S230I) in a patient with breast cancer using exome sequencing of the breast tumor and adjacent normal tissue. *CSF2RB* and seven other immune-related hub genes were identified to be molecular signatures in the subtype of triple-negative breast cancer (TNBC), which may be used as therapeutic targets to treat breast cancer. Huang et al. (2020) estimated the prognostic value of colony-stimulating factors (CSFs) and colony-stimulating factor receptors (CSFRs) through studies across 24 solid cancer types. These studies revealed that in nine (53%) cancer types, one of which is breast invasive carcinoma (BRCA), the high expression of *CSF2RB* is associated with a favorable prognosis (Huang et al., 2020). Analysis of IL-3 and IL-3 receptor subunits (*IL-3RA + CSF2RB*) showed their elevated expression in basal-like and luminal A breast cancers. IL-3, but not IL-3RA or *CSF2RB*, was found to be associated with a poor prognosis in basal-like breast cancer, which supports the role of IL-3 in promoting cancer progression (Thompson et al., 2024). Moreover, histological analysis of IL-3, IL-3RA, and *CSF2RB* expression in healthy breast tissue, primary tumor tissue, and metastatic tissue suggested their potential use as biomarkers in TNBC (Koni et al., 2022). Upregulation of *CSF2RB—*among other genes in basal-like breast cancer with RYR2 and AHNAK mutations*—*has been associated with a good prognosis and correlated with immune infiltrations within tumors (Cimas et al., 2020).

The present study is an attempt to understand the role of *CSF2RB* in breast cancer by collecting omics data from cancer genomic databases such as The Cancer Genome Atlas (TCGA), cBioPortal, TIMER2.0, GEPIA, and UALCAN. The data include gene expression profiles, mutations, methylation patterns, survival outcomes, and immune infiltration related to *CSF2RB*, specifically in breast cancer, and are compared to acute myeloid leukemia as a control since the role of *CSF2RB* in AML is well-established.

## Materials and methods

The significance of bioinformatics methods and tools has become increasingly evident with advances in technology and the accumulation of genome-scale, high-throughput data from genomics, transcriptomics, and proteomics. To analyze the data obtained from experiments of cancer research, bioinformatics scientists have developed different types of computational tools, algorithms, and data structures (Mayakonda et al., 2018; Huang et al., 2024). Moreover, several databases and web-based interactive portals have been developed to access, explore, and analyze the vast amount of cancer genomic and clinical data collected from large-scale studies (TCGA, cBioPortal, TIMER, GEPIA, and UALCAN). These databases and web-portals have been extensively used in the current manuscript; therefore, they are described in more detail below.

### TCGA database

In TCGA (https://portal.gdc.cancer.gov/), approximately 20,000 primary cancer cases and matched normal samples covering 33 cancer types were molecularly described as part of a groundbreaking cancer genomics program. Initiated in 2006, this collaborative endeavor between the NCI and the National Human Genome Research Institute brought together scientists from many universities and disciplines to support cancer researchers in understanding the disease, its clinical development, and its response to treatment. Mutation analysis of the *CSF2RB* gene coding region, specified by the mutation consequence type and the protein domain encoded by the *CSF2RB* gene, was carried out using the protein paint tool. The OncoMatrix tool was used for mutation analysis using the *CSF2RB* coding sequence of 515 cases of breast cancer patients.

### cBioPortal database

cBioPortal (https://www.cbioportal.org/) is a web-based platform for interactively exploring multidimensional cancer genomics datasets. It was originally developed at the Memorial Sloan Kettering Cancer Center (MSK), and the Center for Molecular Oncology at MSK currently hosts the public cBioPortal website. The platform is maintained by a multi-institutional team, including MSK, the Dana-Farber Cancer Institute, the Princess Margaret Cancer Center in Toronto, Children’s Hospital of Philadelphia, Caris Life Sciences, The Hyve, and SE4BIO in the Netherlands, and Bilkent University in Ankara, Turkey. We utilized cBioPortal to analyze the genetic changes and alteration frequencies of the *CSF2RB* gene across breast cancer subtypes in the breast invasive carcinoma (TCGA, Firehose Legacy) dataset. We also studied the mRNA expression of the *CSF2RB* gene across breast cancer subtypes and carried out survival analysis comparing patients with and without *CSF2RB* alterations using the Kaplan–Meier curves.

### TIMER2.0 database

TIMER2.0 (https://timer.comp-genomics.org/timer/) is a comprehensive source for the systematic examination of immune cell infiltration across various types of cancer. It allows users to dynamically produce high-quality visualizations to investigate the immunological, clinical, and genomic aspects of tumors. It also provides the estimates of immune cell abundance using several immune deconvolution algorithms. We performed different *in silico* analyses using the TIMER2.0 database such as the genetic change analysis of *CSF2RB* using the mutation module, differential expression analysis of the *CSF2RB* gene across different types of TCGA tumors and paired normal tissue using the Gene_DE module, subtype-specific differential expression of *CSF2RB* in breast invasive carcinoma using the Gene_Mutation module, correlation analysis between *CSF2RB* expression and immune infiltration levels across diverse cancer types, visualized as a heatmap using the gene module, and survival analysis using Kaplan–Meier curves generated using the Gene_Outcome module.

## GEPIA

GEPIA (https://gepia.cancer-pku.cn/) was developed by Zefang Tang, Chenwei Li, and Boxi Kang of Zhang Lab, Peking University. It is an interactive web server that uses a standard processing pipeline to analyze the RNA sequencing expression data of 8,587 normal samples and 9,736 tumor samples from the TCGA and GTEx projects. Tumor/normal differential expression analysis, cancer type or pathological stage-specific profiling, patient survival analysis, comparable gene detection, and correlation analysis are some of the customizable features offered by GEPIA. We used GEPIA to compare the *CSF2RB* gene expression between breast invasive carcinoma and acute myeloid leukemia and analyze the correlation between the pathological stage of breast invasive cancer and the expression of the *CSF2RB* gene. We carried out a heatmap of the overall survival of the *CSF2RB* gene in breast invasive carcinoma and acute myeloid leukemia.

### UALCAN database

UALCAN (https://ualcan.path.uab.edu/) is a comprehensive resource for OMICS data analysis related to cancer. It uses CSS and JavaScript to create excellent graphics. We analyzed the *CSF2RB* transcript expression per breast invasive carcinoma subclass, performed a pan-cancer analysis of *CSF2RB* expression across normal and tumor samples of TCGA cancer samples, and analyzed *CSF2RB* expression across TCGA tumor samples using UALCAN. We analyzed the *CSF2RB* promoter methylation level in breast invasive carcinoma samples from TCGA and among the major subclasses based on the patient’s race and the pathological stage of breast invasive carcinoma per TCGA sample. We estimated the effect of *CSF2RB* expression on breast invasive carcinoma survival probability.

## Results

### Pan-cancer gene expression of *CSF2RB*

**FIGURE 1:**
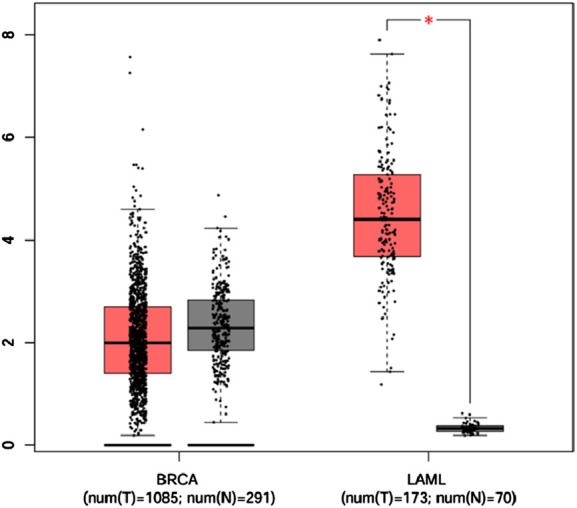
Box plot of *CSF2RB* gene expression comparison between breast invasive carcinoma (291 normal samples and 1,085 tumor samples) and acute myeloid leukemia (70 normal samples and 173 tumor samples) using the GEPIA cancer database that collects data from TCGA and GTEx. The expressions are measured by log_2_ (TPM+1), and expressions of the *CSF2RB* gene in BRCA are slightly higher in normal samples (gray box) than those in tumor samples (red box) but not statistically significant. In the context of AML, the expression of *CSF2RB* in tumor samples (red box) remained significantly higher than that in normal samples (gray box). The star indicates highly significant results.

**FIGURE 2:**
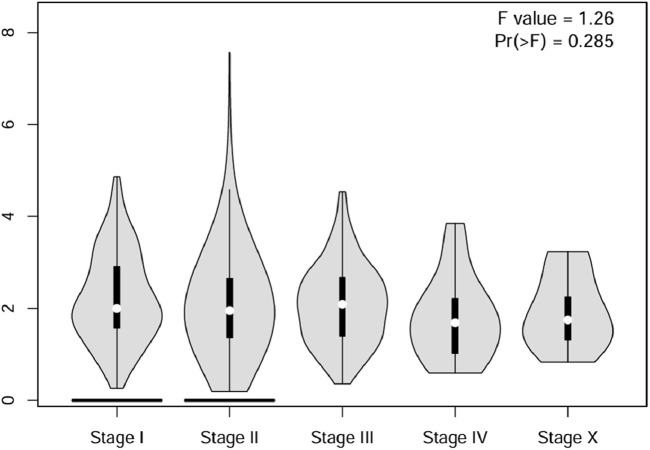
Using the GEPIA cancer database, a violin plot of the *CSF2RB* gene expression across pathological stages of breast invasive carcinoma was plotted. To analyze the differential gene expression of the *CSF2RB* gene, one-way ANOVA was used, and a *p*-value of 0.285 shows that there is no correlation between the pathological stage of breast invasive cancer and the expression of the *CSF2RB* gene.

**FIGURE 3:**
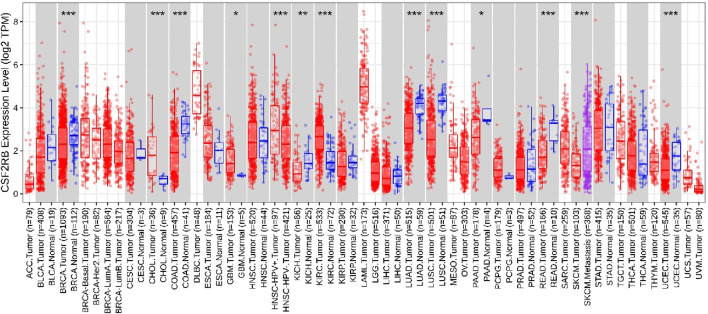
Differential expression analysis using the TIMER2.0 database (Gene_DE module) of the *CSF2RB* gene (log_2_ TPM) across different types of TCGA tumors and paired normal tissue. The stars represent the significance of the results and is calculated using the Wilcoxon test (*: *p*-value <0.05; **: *p*-value <0.01; ***: *p*-value <0.001); data are presented using a box plot. *CSF2RB* gene expression is downregulated in BRCA tumor tissue (number of sample = 1,093) compared to that in normal tissue (number of sample = 112) with *p*-value <0.001. *CSF2RB* gene expression in BRCA-basal (number of sample = 190), BRCA-Her2 (number of sample = 82), BRCA-LumA (number of sample = 564), and BRCA-LumB (number of sample = 217) remained approximately 2–3 (log_2_ TPM). *CSF2RB* gene expression in AML tumor tissue (number of sample = 173) remained approximately 5 (log_2_ TPM), which indicates higher expression of the *CSF2RB* gene in AML tumor tissues than in breast cancer subtypes.

**FIGURE 4:**
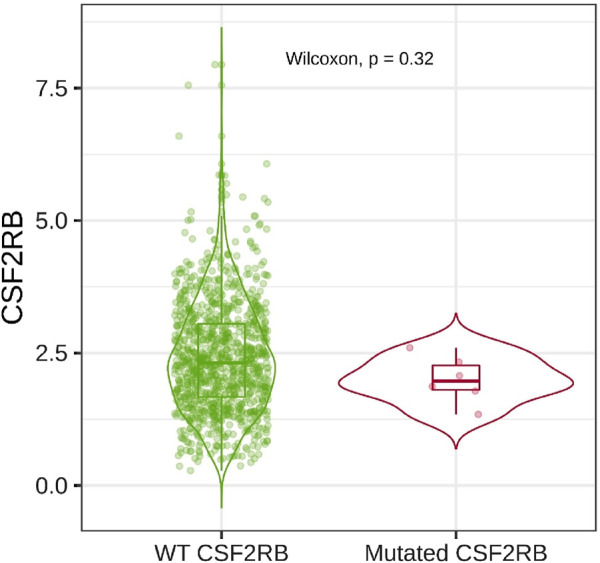
Box plot was generated using the Gene_Mutation module in the TIMER2.0 database to obtain the differential expression of *CSF2RB* in breast invasive carcinoma (number of samples = 1,017; 6 with mutation). Wild-type (WT) *CSF2RB* shows higher expression than the mutated *CSF2RB*, but their expression difference is not statistically significant (*p*-value = 0.32).

**FIGURE 5:**
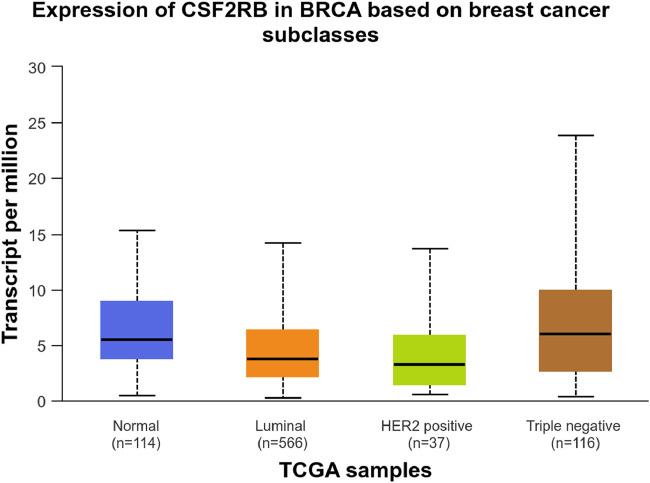
UALCAN database analysis of *CSF2RB* transcript expression per breast invasive carcinoma subclass. Luminal (number of samples = 566) and HER2-positive (number of samples = 37) breast cancer samples showed lower expression of *CSF2RB* than the normal samples (number of samples = 114). TNBC samples (number of samples = 116) showed higher expression than the normal samples, with a *p*-value of 0.032.

**FIGURE 6:**
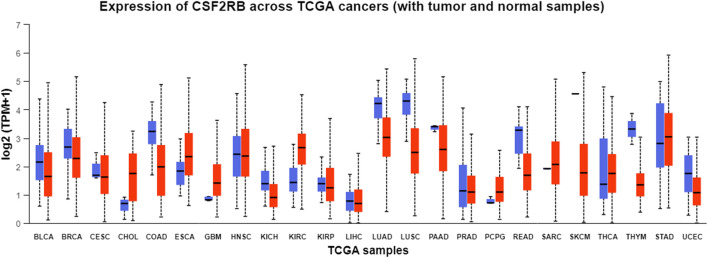
Pan-cancer analysis of *CSF2RB* expression across normal and tumor samples from TCGA program using the UALCAN database. Blue boxes refer to the normal samples, and red boxes refer to the tumor samples. Tumor samples of breast invasive carcinoma (median = 2.297) show lower expression than the paired normal samples (median = 2.699).

**FIGURE 7:**
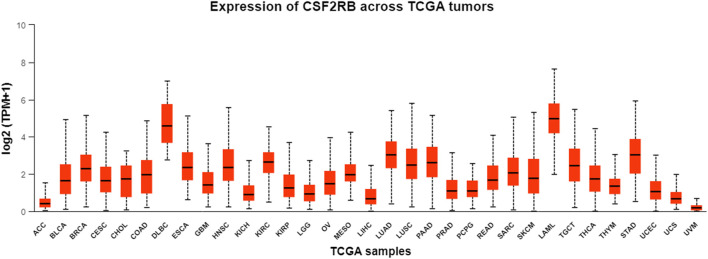
Bar plot of *CSF2RB* expression across TCGA tumor samples using the UALCAN database. The y-axis represents the expression value by log_2_ (TPM+1), and the x-axis represents different TCGA tumor types. Median expression of *CSF2RB* in breast invasive carcinoma (BRCA) is 2.29, and that in AML is 4.97.

Analysis of *CSF2RB* gene expression data from the GEPIA cancer database showed its downregulation in BRCA compared to that in paired normal controls (291 normal samples and 1,085 tumor samples). On the other hand, its expression in AML was upregulated compared to that in paired normal controls (70 normal samples and 173 tumor samples). *CSF2RB* gene expression in AML was found to be very high compared to that in BRCA (Figure 1). Furthermore, we used the same database to investigate the impact of *CSF2RB* expression on the pathological stage of BRCA and found no correlation between the two parameters (Figure 2). Next, the TIMER2.0 database was used to perform expression analysis of the *CSF2RB* gene across different types of TCGA tumors and paired normal tissue. Results showed less or comparable expression of *CSF2RB* in BRCA tumors overall (n = 1,093), BRCA-basal (n = 190), BRCA-Her2 (n = 82), BRCA-LumA (n = 564), and BRCA-LumB (n = 217) compared to that in BRCA normal tissues (n = 112) (Figure 3). Moreover, the highest expression of *CSF2RB* was observed in AML tumor tissue (n = 173). In addition, by analyzing 1,017 BRCA samples in the TIMER2.0 gene mutation database, we observed higher expression of wild-type *CSF2RB* than mutated *CSF2RB* (Figure 4). Finally, the UALCAN database analysis for *CSF2RB* transcript expression in each BRCA sub-class showed downregulation in luminal (n = 566) and HER2-positive (n = 37) subtypes compared to that in paired normal samples (n = 114), and upregulation in TNBC (n = 116) was also noted (Figure 5). Finally, we sought to analyze *CSF2RB* expression across diverse cancer types, including tumor and normal samples of the TCGA program, using the UALCAN database (Figure 6). As illustrated in Figure 6, *CSF2RB* expression was found to be downregulated in BRCA tumor samples compared to that in paired normal samples and upregulated in AML compared to that in its normal counterpart. Figure 7 is similar to Figure 6 but only shows tumor samples. Figure 7 presents the *CSF2RB* expression across different cancer types in only tumor tissues, with a relatively higher expression in DLBC and AML.

### Mutations of CSF2RB at the protein level

**FIGURE 8:**
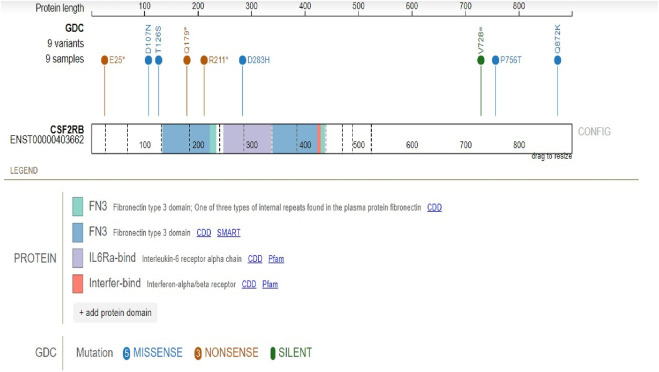
Mutation analysis of the *CSF2RB* gene coding region specified by the mutation consequence type and protein domain encoded by the *CSF2RB* gene. Analysis was performed using 462 cases from the TCGA database using the protein paint tool. Each color of the lollipop represents the mutation type (silent, missense, and nonsense), and the color of the bands reflects different types of encoded protein domains.

Protein domains encoded by *CSF2RB* are the fibronectin type-3 domain, interleukin-6 receptor alpha chain domain, and interferon-alpha/beta receptor domain. The results obtained using the “protein paint tool” showed five missense mutations, three nonsense mutations, and one silent mutation (Figure 8). Therefore, according to the TCGA platform, the mutation frequency of *CSF2RB* computes to 1.94%.

**FIGURE 9:**
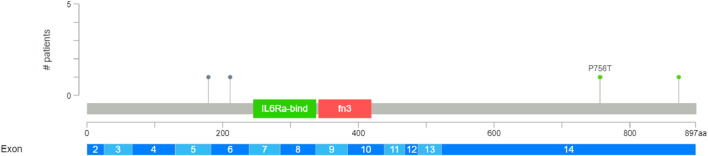
Breast invasive carcinoma study conducted by TCGA, Firehose Legacy, across 1,108 samples and 1,101 patients. Mutation diagram generated using cBioPortal shows colored circles reflecting the corresponding mutation types. This diagram displays four mutations in the *CSF2RB* gene among all samples; two of them are nonsense mutations (AA change: Q179* and R211*, respectively) and two are missense mutations (AA change: P756T and Q872K, respectively). The frequency of somatic mutations among patients is 0.4%.

To investigate the frequency of somatic mutations in *CSF2RB* among patients, we analyzed data from the cBioPortal database based on the TCGA Firehose Legacy study, which included 1,108 samples obtained from 1,101 patients. Four mutations were present among all samples, two of which are nonsense mutations and two are missense mutations with a frequency of approximately 0.4% among all samples (Figure 9).

### *CSF2RB* somatic mutations across breast cancer subtypes and other cancer types

**FIGURE 10:**
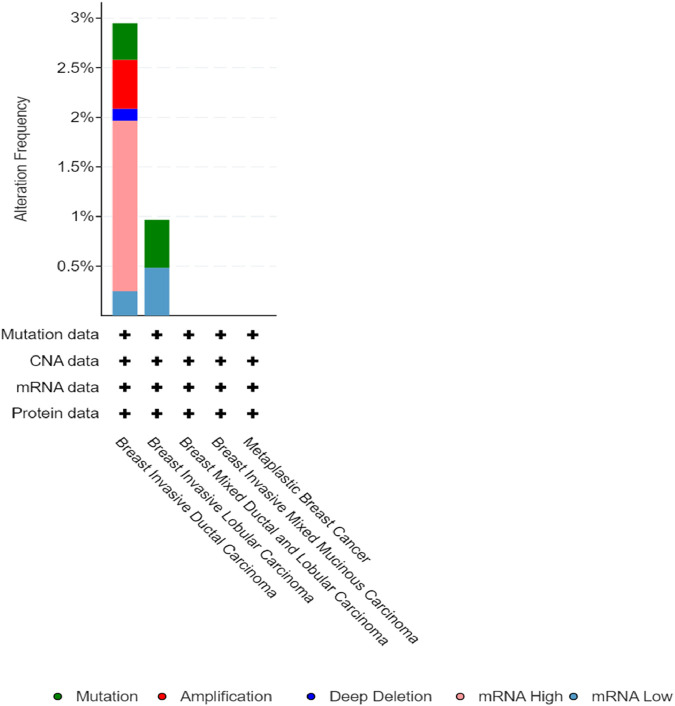
Alteration frequency of the *CSF2RB* gene per breast cancer subtype extracted from the cBioPortal database indicates that breast invasive ductal carcinoma has the highest alteration frequency of approximately 3%, and breast invasive lobular carcinoma has approximately 1%. The type of alteration is represented by different colors, as shown in the figure above. The other subtypes did not show any alteration of the *CSF2RB* gene in particular.

**FIGURE 11:**
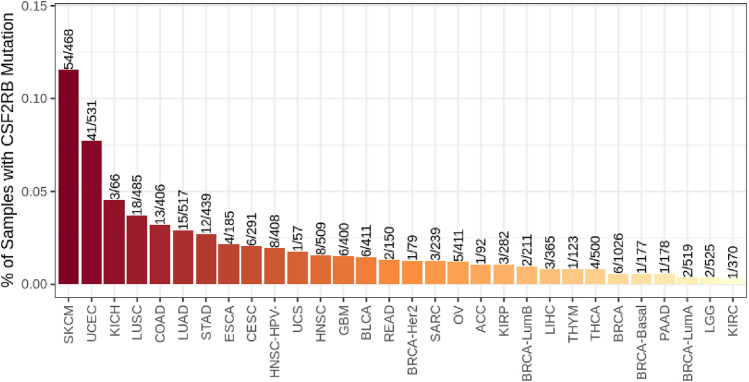
Bar plot of the *CSF2RB* gene mutation frequency among TCGA cancer types. BRCA-basal (number of sample = 177), BRCA-Her2 (number of sample = 79), BRCA-LumA (number of sample = 519), and BRCA-LumB (number of sample = 211) show 0.56%, 1.26%, 0.38%, and 0.94% *CSF2RB* mutation frequencies, respectively. In aggregate, BRCA (number of sample = 1,026) shows 0.58% mutation frequency. The analysis was performed using the TIMER2.0 database mutation module.

Breast invasive ductal carcinoma had the highest mutation frequency, followed by breast invasive lobular carcinoma, according to the study carried out on 1,100 samples of BRCA by TCGA, Firehose Legacy (Figure 10). The TIMER2.0 database mutation module showed that 0.5% of BRCA samples have *CSF2RB* mutation, while skin cutaneous melanoma (SKCM) showed the highest rate of 11.5% of samples mutated for this gene (Figure 11).

### Methylation of the *CSF2RB* gene/promoter across cancers

**FIGURE 12:**
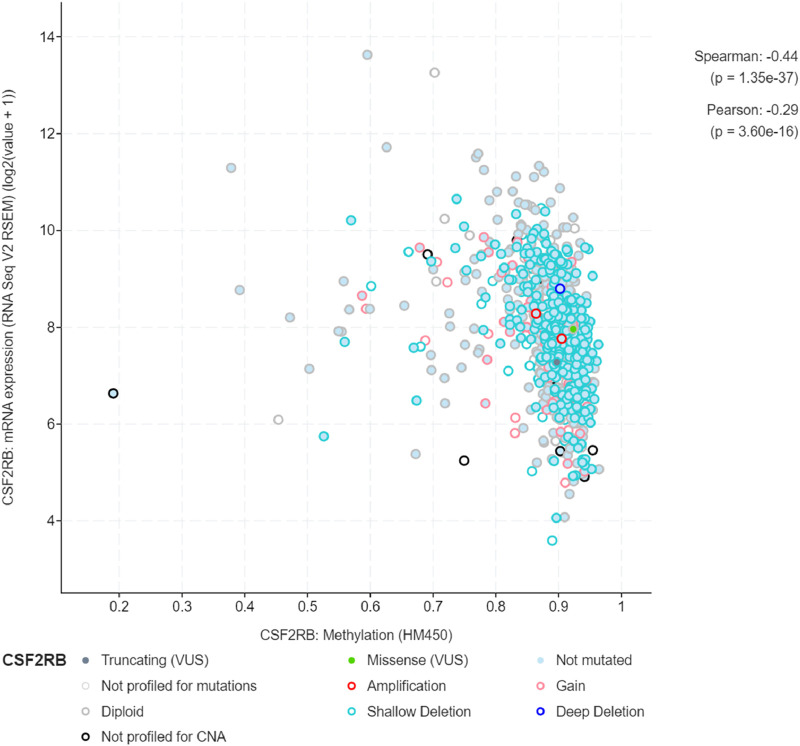
A total of 785 samples from the breast invasive carcinoma (TCGA, Firehose Legacy) study were analyzed using the cBioPortal database to compare *CSF2RB* mRNA expression and methylation levels across samples. Spearman and Pearson correlation analyses indicate a negative correlation between these two variables. As the CSF2RB transcript expression increases, the methylation of CSF2RB decreases and vice-versa. Each colored circle corresponds to a specific type of *CSF2RB* alteration.

**FIGURE 13:**
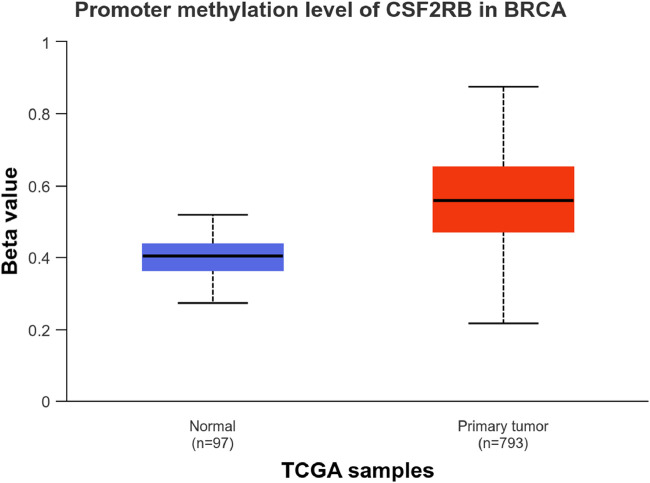
*CSF2RB* promoter methylation level in breast invasive carcinoma per TCGA samples; the beta value (y-axis) reflects the level of methylation from 0 (un-methylated) to 1 (fully methylated). The beta value for hypermethylation is 0.5–0.7, and that for hypomethylation is 0.25–0.3. Primary tumor samples are hypermethylated (median = 0.558), and normal samples are neither hyper- nor hypo-methylated (median = 0.403). Analysis was carried out using the UALCAN database.

**FIGURE 14:**
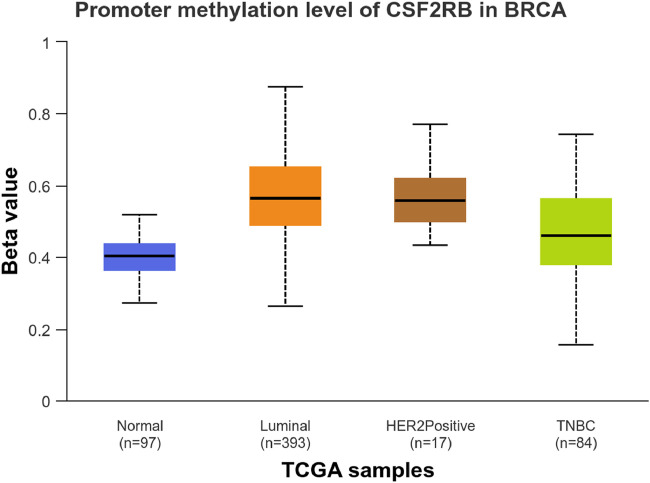
*CSF2RB* promoter methylation level among the major subclasses of breast invasive carcinoma per TCGA samples using the UALCAN database; the beta value reflects the level of methylation from 0 (unmethylated) to 1 (fully methylated). The beta value for hypermethylation is 0.5–0.7, and the beta value for hypomethylation is 0.25–0.3. Normal samples and triple-negative breast cancer samples are neither hyper- nor hypo-methylated (median of 0.403 and 0.459, respectively), while luminal breast cancer and HER2+ breast cancer are hyper-methylated (median of 0.564 and 0.559, respectively).

**FIGURE 15:**
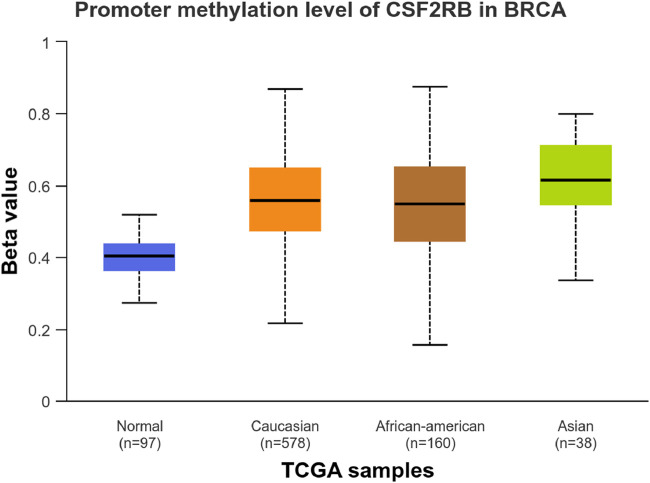
*CSF2RB* promoter methylation level based on the patient’s race having breast invasive carcinoma per TCGA samples using the UALCAN database; the beta value reflects the level of methylation from 0 (un-methylated) to 1 (fully methylated). The beta value for hyper-methylation is 0.5–0.7, and the beta value for hypo-methylation is 0.25–0.3. Normal samples are neither hyper nor hypo-methylated (median = 0.403). Meanwhile, samples of Caucasian, African–American, and Asian patients are hypermethylated (median of 0.557, 0.549, and 0.615, respectively).

**FIGURE 16:**
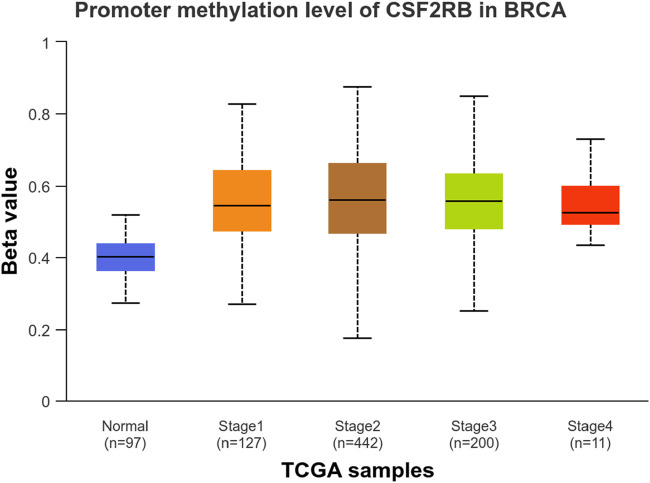
UALCAN database analysis of the *CSF2RB* promoter methylation level based on breast invasive carcinoma stages per TCGA samples. The beta value reflects the level of methylation from 0 (un-methylated) to 1 (fully methylated). The beta value for hypermethylation is 0.5–0.7, and the beta value for hypomethylation is 0.25–0.3. Normal samples are neither hyper- nor hypo-methylated (median = 0.403). BRCA stage 1–4 samples are hypermethylated, with only slight differences in the level of methylation; the methylated median values are 0.546, 0.56, 0.557, and 0.526 for stages 1, 2, 3, and 4, respectively.

We observed an inverse correlation between *CSF2RB* mRNA expression and its methylation (Figure 12). Our analysis revealed hypermethylation of tumor samples (n = 793) compared to normal samples for BRCA patients (Figure 13). Moreover, luminal and HER2+ breast cancers showed hypermethylation, and normal samples and triple-negative breast cancer samples were neither hyper- nor hypo-methylated (Figure 14). With respect to the patients’ ethnicity, different methylation levels were observed in the *CSF2RB* promoter region (Figure 15). However, different stages of BRCA (stages 1–4) showed hypermethylation of the *CSF2RB* promoter (Figure 16).

### Prognostic effect of *CSF2RB* across cancers

**FIGURE 17:**
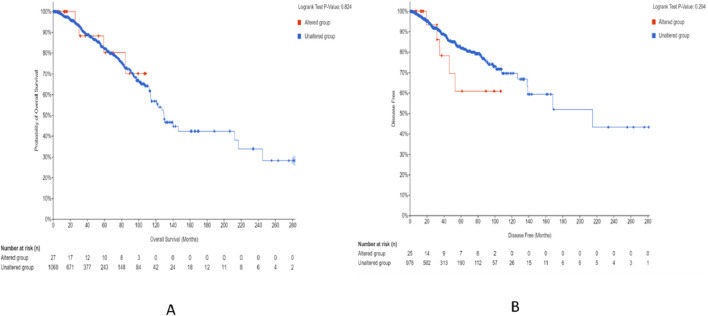
Survival study on breast invasive carcinoma that was carried out by TCGA and Firehose Legacy using 1,101 patients and 1,108 samples. Survival analysis of patients with altered (red line) and unaltered (blue line) *CSF2RB* gene using the Kaplan–Meier curve. **(A)** Probability of overall survival (y-axis) across time in months (x-axis) was plotted; in this graph, there are 27 altered groups and 1,068 unaltered groups, with log-rank 0.824 *p*-value. There are no obvious variations between the two groups. **(B)** Disease-free survival probability among altered (25 cases) and unaltered (978 cases) groups with log-rank 0.294 *p*-value. Over time, patients in the altered group exhibited a lower disease-free survival probability than those in the unaltered group.

**FIGURE 18:**
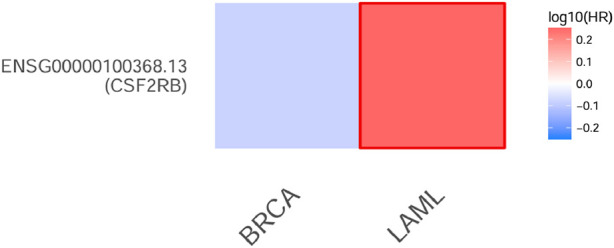
Heatmap of overall survival (per months) analysis of the *CSF2RB* gene in breast invasive carcinoma and acute myeloid leukemia using the GEPIA 2 database. Log10 HR (hazards ratio) is employed as a measurement. Red represents poor prognosis, whereas blue represents good prognosis, and the intensity of the color is correlated with the HR value. The boundaries surrounding the color indicate significant *p*-value <0.05. The *CSF2RB* gene has a negative prognosis in case of AML and probably (statistically not significant) good prognostic effect in case of BRCA.

**FIGURE 19:**
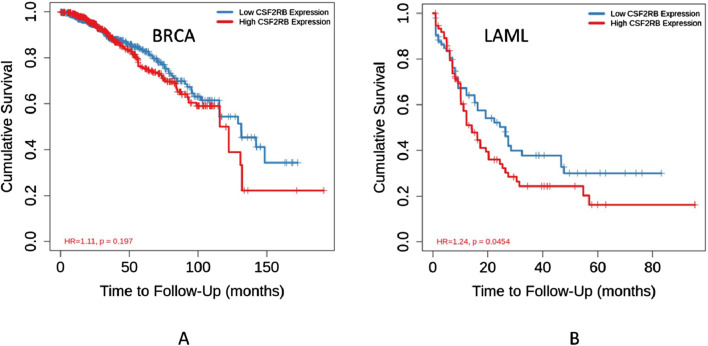
Kaplan–Meier curve of cumulative survival of 1,100 samples with low or high *CSF2RB* gene expression was generated using the Gene_Outcome module by the TIMER2.0 database. **(A)** Cumulative survival of BRCA samples with low or high *CSF2RB* expression is not statistically significant. **(B)** AML samples with high *CSF2RB* expression showed a lower survival rate than samples with lower *CSF2RB* expression.

**FIGURE 20:**
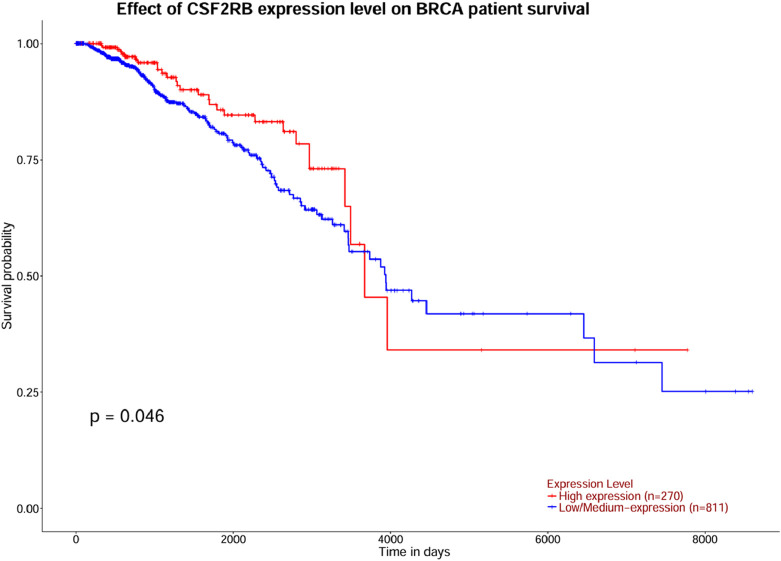
Survival analysis was carried out using the UALCAN database for BRCA patients with high and low *CSF2RB* expression. The survival probability of patients with high *CSF2RB* expression is statistically different than that of patients with low *CSF2RB* expression (p = 0.046).

The survival analysis was carried out using 1,101 patients and 1,108 samples (from the TCGA Firehose Legacy) of breast invasive carcinoma collected through cBioPortal. Figures 17A, B show that the overall survival and disease-free survival of BRCA patients are not affected by the alteration status of the *CSF2RB* gene. A comparison between the overall survival of the *CSF2RB* gene in breast invasive carcinoma and acute myeloid leukemia showed that the *CSF2RB* gene has a negative prognosis in cases of AML and a good prognostic effect in cases of BRCA (Figure 18). Another comparison of cumulative survival of patients (1,100 samples) with low or high *CSF2RB* expressions for breast invasive carcinoma and acute myeloid leukemia conducted by the TIMER2.0 database shows that the cumulative survival of BRCA samples remained the same until approximately 120 months, when the samples with high *CSF2RB* expression showed a decline in the cumulative survival rate (statistically not significant, *p* = 0.197), while AML samples with high *CSF2RB* expression showed a decline in the cumulative survival rate (statistically significant, *p* = 0.0454) after a short time (Figure 19). Survival probability data obtained from the UALCAN database for BRCA samples (Figure 20) showed a distinct difference in survival between patients with high and low *CSF2RB* expression. The results presented in Figures 19, 20 appeared to be contradictory.

### Immune infiltration level in diverse cancer types and *CSF2RB* expression

**FIGURE 21:**
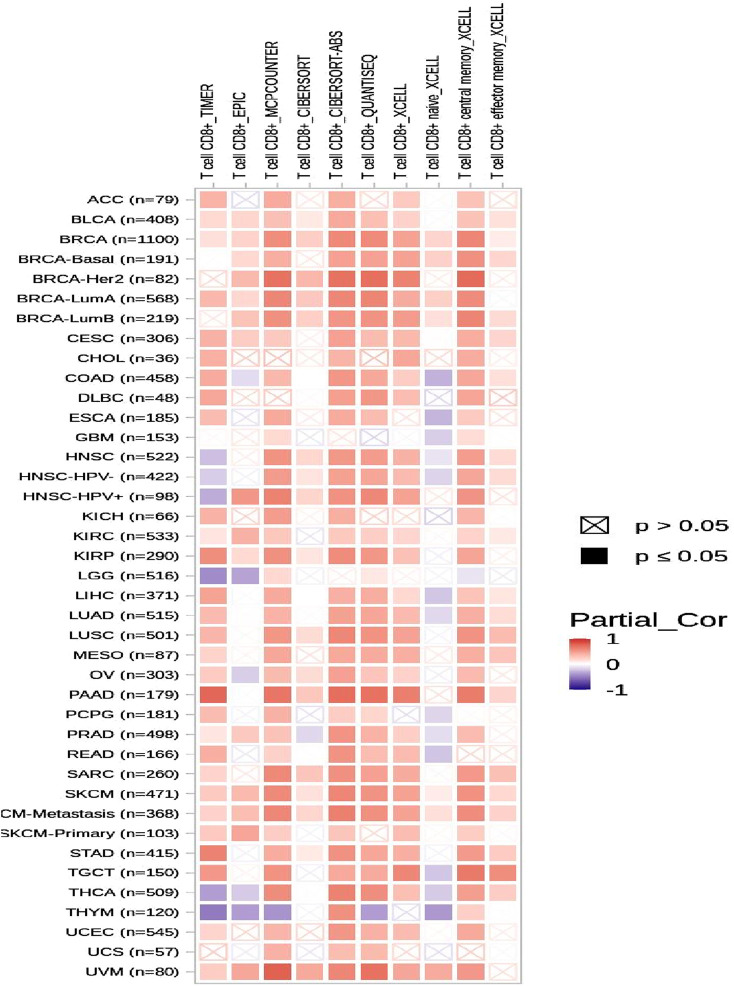
Heatmap of correlation analysis of the immune infiltration level in diverse cancer types and *CSF2RB* expression based on the TIMER2.0 database using the gene module. Red represents positive correlation, and blue represents negative correlation; the stronger the association, the darker the color. Significant *p*-values (≤0.05) are represented by shaded squares, and non-significant *p*-values (>0.05) are represented by crossed squares. BRCA (number of samples = 1,100), BRCA-basal (number of samples = 191), BRCA-Her2 (number of samples = 82), BRCA-LumA (number of samples = 568), and BRCA-LumB (number of samples = 219) showed high correlation between CD8 T-cell infiltration level and *CSF2RB* gene expression level.

To assess the correlation between *CSF2RB* expression and the degree of immune infiltration in various cancer types, the TIMER database was examined (Figure 21). The results demonstrated that the expression of *CSF2RB* was correlated to the CD8 T-cell infiltration level in BRCA (number of samples = 1,100), BRCA-basal (number of samples = 191), BRCA-Her2 (number of samples = 82), BRCA-LumA (number of samples = 568), and BRCA-LumB (number of samples = 219). Immune cells that migrate from the circulation into the tumor are known as tumor-infiltrating immune cells and represent a key component of the tumor microenvironment. These cells include natural killer cells, macrophages, T cells, and B cells (Li et al., 2022). The interactions between these cells and cancer cells through signaling pathways may lead to cancer progression and therapy outcomes (Zou et al., 2021). Cytotoxic T cells (CD8) destroy cancer cells, and helper T cells (CD4^+^) provide support to the immune cells. B cells produce antibodies against cancer cells. Macrophages attack cancer cells and, in some cases, enhance tumor growth. Dendritic cells regulate immune response (Li et al., 2022). T regulatory cells (Tregs) (Zhu et al., 2022), alternatively activated macrophages (M2 macrophages) (Hao et al., 2012), and tumor-associated dendritic cells (TADCs) (Hsu et al., 2018) can contribute to tumor progression and metastasis. Researchers can develop more effective therapies and enhance patient prognosis by examining the patterns and types of immune cells found within the tumor microenvironment (TME) (Zou et al., 2021). If the gene expression is correlated with specific types of immune cells in the TME, it indicates the potential prognostic value of that gene in that cancer type (Hu et al., 2023).

## Discussion

Analyses of the cancer genomics data related to the *CSF2RB* gene/protein from different databases revealed some striking results that need to be discussed in order to draw the right conclusion from this study. The *CSF2RB* gene is expressed in tumors across all cancer types available from the TCGA study (Figure 7). In addition, the expression data are available for normal and matched tumor samples from all cancer types (Figures 3, 6). In BRCA, the median expression of the *CSF2RB* gene is higher in normal samples than in the matched tumor samples (Figures 1, 3, 6). These results were obtained from GEPIA, TIMER2.0, and UALCAN databases, respectively. The result from GEPIA (Figure 1) was not statistically significant. In contrast, the result (Figure 3) obtained from the TIMER2.0 database (hosting only TCGA data) showed that BRCA normal samples had significantly higher expression of *CSF2RB* than BRCA tumor samples (Figure 3). However, in all these results (Figures 1, 3, 6), *CSF2RB* gene expression is higher in normal than in the tumor tissues in the context of BRCA. This observation is also supported by other studies. Analysis of the mutational effect of *CSF2RB* on its expression revealed no statistically significant difference between the expression levels of the wild-type and mutated *CSF2RB* gene (Figure 4). Moreover, the expression of this gene is not associated with the pathological stages of BRCA tumor samples (Figure 2). However, the expression differs across different subtypes of breast cancer, with luminal and HER2+ subtypes expressing lower and TNBC expressing higher than normal samples (Figure 5). This observation is not surprising because of the heterogeneous nature of the breast cancer histological/molecular subtypes. Together, these results indicate that the *CSF2RB* gene potentially plays i) a protective or tumor-suppressive role in luminal and HER2+ subtypes and ii) an oncogenic role in triple-negative breast cancers (TNBC). In contrast, the *CSF2RB* gene is significantly more highly expressed in tumors than in normal samples in AML (Figure 1). In addition, the highest expression of this gene was observed in AML and diffuse large B-cell lymphoma (DLBC), among other cancer types (Figures 3, 7). These results suggest the potential oncogenic role of *CSF2RB* in hematological cancer. Moreover, this association has been recently established and published in various research articles.

Mining TCGA data using the “protein paint tool” for coding mutations in the *CSF2RB* gene resulted in a total of nine mutations, including five missense, three nonsense, and one silent mutation (Figure 8). Exploring cBioPortal (hosting TCGA data across 1,108 samples/1,101 patients) resulted in a total of four somatic mutations in the *CSF2RB* gene, with a frequency of approximately 0.4% among all samples (Figure 9). Among different subtypes of breast cancer, invasive ductal carcinoma showed higher alteration frequency than invasive lobular carcinoma (Figure 10). Across different cancer types, the mutation frequency of the *CSF2RB* gene is the highest in skin cutaneous melanoma (11.5%) and ranges from 0.38% to 1.26% in breast cancer subtypes (Figure 11). In summary, somatic mutations in *CSF2RB* are not abundant in BRCA, suggesting a limited or inconsistent role as a driver in the tumor progression of breast carcinoma.

The study of *CSF2RB* mRNA expression and its DNA methylation revealed a negative correlation, with a Pearson correlation coefficient of −0.29 and a Spearman correlation coefficient of −0.44 (Figure 12). DNA methylation impacts the gene expression pattern, and this phenomenon was discovered half a century ago (McGhee and Ginder, 1979; Jones and Taylor, 1980). The negative correlation between mRNA expression and DNA methylation has been instrumental in identifying several key genes in different diseases, such as obesity (Chen et al., 2021). DNA methylation plays a profound role in mammalian development (Smith and Meissner, 2013) and is also implicated in various diseases (Robertson, 2005). As expected, the promoter of the *CSF2RB* gene is hypermethylated in tumor tissues, and the methylation level is higher than that in normal tissues (Figure 13). This methylation difference explains the reason behind the higher gene expression of *CSF2RB* in normal tissues than that in tumor tissues, as shown in Figure 1. Promoter methylation of *CSF2RB* across different BRCA subtypes varies greatly, with luminal and HER2+ being hypermethylated and TNBC not being hypermethylated (Figure 14), which explains the gene expression pattern of this gene across different subtypes, as shown in Figure 5. Promoter methylation across different ethnic populations shows that the *CSF2RB* promoter is hypermethylated in breast tumors of Caucasian, African-American, and Asian populations (Figure 15). Figure 16 shows no difference in promoter methylation across BRCA stages, and this result explains the gene expression behavior across stages, as shown in Figure 2. In conclusion, the methylation pattern of the *CSF2RB* gene promoter across different breast cancer subtypes, stages, and ethnicities agrees very well with the gene expression pattern in an inverse manner.

Survival analyses have concluded that overall and disease-free survival probabilities are not significantly different between BRCA patients with or without *CSF2RB* genetic alteration (Figure 17). It means that *CSF2RB* does not have a significant impact on the pathobiology of breast carcinoma. However, the disease-free survival probability of patients with the altered *CSF2RB* gene remains lower than that of patients with the unaltered gene (Figure 17B). Comparison of overall survival between *CSF2RB-*altered and unaltered patient groups in BRCA and AML showed that the *CSF2RB* alterations are associated with a poorer prognosis in the case of AML but with a favorable prognosis in BRCA (Figure 18). Survival analysis based on *CSF2RB* expression levels shows similar results (Figure 19), where differences in *CSF2RB* expression do not significantly impact cumulative survival in BRCA patients (Figure 19A) groups but do have an impact in AML cases (Figure 19B). This conclusion is drawn on the basis of the log-rank *p*-value, which is 0.197 (non-significant) in the case of BRCA and 0.045 (significant) in the case of AML. However, a separate survival analysis of BRCA patients using the UALCAN database indicates that a statistically significantly difference in survival probability between groups with high and low *CSF2RB* expression (Figure 20). At first glance, the results from Figures 19, 20 appear to be contradictory. The reasons could be minor differences in the extent of data, such as i) the number of patients, ii) follow-up time, and iii) the expression variations across different assay platforms. At the end, the correlation analysis of *CSF2RB* expression and immune infiltration levels shows that there is a positive correlation of CD8 T-cell infiltration level and *CSF2RB* expression, stating the positive prognostic value of *CSF2RB* in BRCA patients and different subtypes (Figure 21). In general, BRCA patients have a positive correlation between *CSF2RB* expression and CD8^+^ T-cell infiltration level from different sources, with a *p-*value <0.05. However, we observe some insignificant (p > 0.05) positive correlations when samples are stratified into BRCA subtypes. The possible explanations for these discrepancies could be divergent data sources and their differences in data processing across different platforms.

The role of *CSF2RB* and other similar receptors in hematological or blood cancers was established in recent decades through several scientific research studies using hematological cell lines, primary cells, animal models, and human patients (Watanabe-Smith et al., 2016; Liongue and Ward, 2014; Maxson et al., 2013). *CSF2RB* mutations or alterations in signaling pathways and expression can contribute to cancer progression (Watanabe-Smith et al., 2016; Watanabe-Smith et al., 2015; Kassem et al., 2018). Other studies have shown that high *CSF2RB* expression is associated with an unfavorable prognosis in bladder and esophageal cancers, whereas it is related to a favorable prognosis in breast cancer, cervical squamous cell carcinoma and endo-cervical adenocarcinoma (CESC), and colon adenocarcinoma (Huang et al., 2020). The expression of *CSF2RB* in lung adenocarcinoma was lower in the tumor sample than in the normal sample, and low expression was related to poor survival (Zhu et al., 2022). *CSF2RB* expression level was also positively related to the levels of infiltrating CD4^+^ T cells, macrophages, NK cells, and monocytes in LUAD (Zhu et al., 2022). Similarly, our results showed that *CSF2RB* expression was correlated to CD8 cell infiltration levels in BRCA and its subtypes, and it could be associated with the immune response in the tumor microenvironment and may lead to immune cell activation or recruitment, which makes it a potential target for breast cancer therapy. *CSF2RB* has a low somatic mutation rate in breast cancer samples/patients, with a frequency of approximately 0.4%. The *CSF2RB* promoter methylation level in tumor samples of breast carcinoma and its subtypes and normal samples revealed that tumor samples are hypermethylated, which can explain the low expression of *CSF2RB* in tumor samples. Normal tissue and triple-negative breast cancer have normal levels of methylation. DNA methylation, an important epigenetic factor, is known to regulate gene expression (Moore et al., 2013).

In conclusion, *CSF2RB* is generally downregulated in breast tumor tissues, but its expression varies across different subtypes. Luminal and HER2+ subtypes exhibit lower expression and TNBC shows higher expression than that of normal tissues (Figure 5). *CSF2RB* does not seem to be a somatic mutational hot spot since the frequency of mutation in samples is very low. Moreover, promoter methylation patterns correspond well with the gene expression profiles across different subtypes of BRCA (Figure 14). The prognostic value of *CSF2RB* is not very clear and statistically significant in the current study (Figures 17–19), except in one experiment using the UALCAN database (Figure 20). Therefore, we find it difficult to conclude that *CSF2RB* could be a prognostic biomarker for BRCA. One of the possible explanations for the ambiguous prognostic effect could be heterogeneity of data sources, and we need to study these effects in each subtype separately. However, functional studies are required to claim *CSF2RB* as a breast cancer hallmark gene or a potential therapeutic and prognostic candidate for breast cancer treatment. Studies involving gene expression, DNA methylation, and integration with survival data for each of the BRCA subtype samples are highly recommended.

## Limitations

The major limitation of the current study is the integration of data from different sources, study designs, assay platforms, and bioinformatics analysis pipelines. These factors might contribute to the visible discrepancies in terms of statistical significance, particularly *p*-values.

## Data Availability

The original contributions presented in the study are included in the article/supplementary material; further inquiries can be directed to the corresponding author.

## Glossary

Aml: Acute myeloid leukemia
ACC: Adrenocortical carcinoma
BLCA: Bladder urothelial carcinoma
LGG: Brain lower grade glioma
BRCA: Breast invasive carcinoma
CESC: Cervical squamous cell carcinoma and endo-cervical adenocarcinoma
CHOL: Cholangiocarcinoma
LCML: Chronic myelogenous leukemia
COAD: Colon adenocarcinoma
CNTL: Controls
ESCA: Esophageal carcinoma
FPPP: FFPE pilot phase II
GBM: Glioblastoma multiforme
HNSC: Head and neck squamous cell carcinoma
KICH: Kidney chromophobe
KIRC: Kidney renal clear cell carcinoma
KIRP: Kidney renal papillary cell carcinoma
LIHC: Liver hepatocellular carcinoma
LUAD: Lung adenocarcinoma
LUSC: Lung squamous cell carcinoma
DLBC: Lymphoid neoplasm diffuse large B-cell lymphoma
MESO: Mesothelioma
MISC: Miscellaneous
OV: Ovarian serous cystadenocarcinoma
PAAD: Pancreatic adenocarcinoma
PCPG: Pheochromocytoma and paraganglioma
PRAD: Prostate adenocarcinoma
READ: Rectum adenocarcinoma
SARC: Sarcoma
SKCM: Skin cutaneous melanoma
STAD: Stomach adenocarcinoma
TGCT: Testicular germ cell tumors
THYM: Thymoma
THCA: Thyroid carcinoma
UCS: Uterine carcinosarcoma
UCEC: Uterine corpus endometrial carcinoma
UVM: Uveal melanoma
